# Effect of Glycerol Stearates on the Thermal and Barrier Properties of Biodegradable Poly(butylene Adipate-Co-Terephthalate)

**DOI:** 10.3390/ma17235732

**Published:** 2024-11-23

**Authors:** Jing Yuan, Xinpeng Zhang, Jun Xu, Jianping Ding, Wanli Li, Baohua Guo

**Affiliations:** 1 Key Laboratory of Advanced Materials (MOE), Department of Chemical Engineering, Tsinghua University, Beijing 100084, China; yuanj22@mails.tsinghua.edu.cn (J.Y.); zhangxp20@mails.tsinghua.edu.cn (X.Z.); 2 Xinjiang Blue Ridge Tunhe Sci. & Tech. Co., Ltd., Changji 831100, China

**Keywords:** biodegradability, barrier properties, poly(butylene adipate-co-terephthalate), glycerol stearates

## Abstract

Two types of glycerol stearates, glycerol monostearate (GMS) and glycerol tristearate (GTS), were added into poly(butylene adipate-co-terephtalate) (PBAT), with the aim to improve their water vapor barrier properties. The effects of the two small molecules on microstructure, chain mobility, and surface hydrophobicity were amply assessed via both experimental and simulation methods. The incorporation of the modifiers at small loadings, 5 wt% of GMS and 1 wt% of GTS, resulted in substantial improvements in water vapor barrier properties, while a further increase in the modifier content resulted in deterioration. The optimal water vapor permeability reached values of 2.63 × 10^−13^ g·cm/(cm^2^·s·Pa) and 6.55 × 10^−13^ g·cm/(cm^2^·s·Pa), which are substantially lower than the permeability, 8.43 × 10^−13^ g·cm/(cm^2^·s·Pa), of neat PBAT. The water vapor permeability of PBAT/GMS blends was also proven to be time-dependent and dramatically decreased with time, mainly due to the migration process of small molecules, forming a waterproof layer. The barrier improvement results are assumed to be related to the hydrophobic effect of glycerol stearate and are largely dependent on the content, polarity, compatibility, and dispersion of modifiers. In addition, the incorporation of modifiers did not largely sacrifice the mechanical strength of PBAT, which is advantageous in mulch film applications.

## 1. Introduction

With the development of the Global Plastics Treaty mandated by the UN to end plastic pollution, the sustainability of plastics is becoming increasingly crucial [1,2]. Traditional plastic films (representatively, polyethylene (PE)) are hard to recycle or degrade, and they cause serious environmental pollution and economic loss. Biodegradable polyesters, as an alternative to traditional film materials, have indispensable applications in single-use materials and agricultural mulch [3,4]. The intrinsic aliphatic ester bonds and hierarchical structure endow them excellent biodegradability, rendering them suitable for solving the current environmental problems introduced by the improper disposal of polymer products [5]. However, various biodegradable polyester challenges emerge in relation to the poor gas/moisture barrier properties, which greatly limit the applications in agricultural mulch film [6]. Biodegradable polyesters such as polybutylene succinate (PBS), polylactide (PLA), polypropylene carbonate (PPC), and especially poly(butylene adipate-co-terephthalate) (PBAT) are more permeable to small molecules than PE, which reveal a worse oxygen-resistant and moisture-resistant effect when used as film materials [7]. In particular, PBAT has been widely used in agricultural mulch due to its balanced mechanical properties, processing ease in film blowing, and good biodegradability [8,9,10], but its low crystallinity and less dense arrangement result in a polymer matrix that has greater free volume and, therefore, poor barrier properties. The oxygen permeability of PBAT is reported to be higher than 4.0 × 10^−11^ g·m/(m^2^·s·Pa) [3,11], which is almost four times higher than that of PLA and much higher than those of polyethylene, poly(ethylene terephthalate), etc. The poor oxygen/moisture isolation and poor water retention capabilities of PBAT limit its application in agricultural mulching.

Due to the multi-hierarchical structures of polymers, including chain mobility (free volume) and crystallinity, the permeation of small molecules is usually complex, especially for water vapor, which can form water trimer, tetramer, or pentamer structures [12]. The permeation of small molecules through a polymer matrix is commonly described via the absorption and diffusion process [6,13], which is often described as a “solution-diffusion” model, and it is expressed as Formula (1) [14] as follows:

(1) P=D·S

where *P* represents permeability; *D* denotes the diffusion coefficient, which describes the kinetic movement of small molecules in the polymer matrix; and *S* is the solubility coefficient, characterizing the dissolution of small molecules to the polymer’s surface.

Based on the above analysis, the incorporation of modifiers into the polymer matrix to either decrease the diffusion or solubility of small molecules provides a methodology for resolving the inferior barrier properties of biodegradable polymers [15,16,17]. On the one hand, many research studies focus on creating a tortuous diffusion pathway that penetrates into the polymer in order to enhance barrier properties, such as by adding flake silicate [18,19] and carbon materials [20], which decrease permeability by decreasing the diffusion coefficient *D*. However, stiff flake silicate or carbon materials largely sacrifice ductility and processing performance. On the other hand, intermolecular interactions in the polymer matrix are also a crucial aspect for the diffusion of penetrates. There are a few research studies utilizing small molecules as modifiers, and the results proved that the introduction of small molecules resulted in a significant change in the physical state of the amorphous phase without detectable changes in the crystalline phase [21,22,23]. This means that crystallinity, as a crucial factor influencing the barrier properties, is barely affected. Taking this into account, regulating the amorphous phase to improve the barrier properties of biodegradable polymers via the incorporation of some specific small molecules is a convenient method.

Solubility, as a significant factor influencing ultimate permeability, especially with respect to polar penetrates such as water vapor, is often overlooked. Therefore, it is important to evaluate weakly polar, small molecules as modifiers that tailor solubility and therefore barrier properties. Glycerol stearates, as commonly used additives in daily chemicals and food processing, are fully non-toxic and eco-friendly [24]. Therefore, decreasing permeability through the incorporation of glycerol stearate to reduce solubility could be a feasible method.

In this study, two weakly polar small molecules, glycerol monostearate and glycerol tristearate, were utilized to improve the water vapor barrier properties of PBAT by reducing the absorption process of penetrates. PBAT/modifier films were fabricated via the melt mixing technique, and the water vapor permeability of different mixtures was analyzed in detail. The effect of the two small molecules on the microstructure, chain mobility, and surface hydrophobicity was also investigated via both experimental and simulation methods.

## 2. Experimental Section

### 2.1. Materials

PBAT (pellet form) was provided by Xinjiang Blueridge Tunhe Polyester Co., Ltd. in Changji, Xinjiang, China (grade: TH801T with a density of 1.20–1.28 g/cm^3^ and melt flow index (MFI) between 2.5 and 4.5 g/10 min measured at 190 °C with a load of 2.16 Kg). Glycerol monostearate (GMS, 95%) was purchased from Meryer Biochemical Technology Co., Ltd. (Shanghai, China), and glycerol tristearate (GTS, 97%) was obtained from Kaiguo Tech. Co., Ltd. (Beijing, China). Other reagents and solvents were of analytical grade and were used as received.

### 2.2. Sample Preparation

PBAT with small molecular compound systems was prepared using the melt mixing technique. PBAT pellets were pre-dried at 60 °C in a vacuum oven overnight before use. The PBAT/glycerol stearate systems containing GMS (1–7 wt%, feeding percentage) or GTS (1–7 wt%, feeding percentage) were prepared through melt blending at 150 °C and 50 rpm for 5 min with a torque rheometer (PolylabOS, Thermo Fisher Scientific Inc., Waltham, MA, USA). The films from all compositions with a thickness of 350–400 μm were created via hot pressing.

### 2.3. Characterization

#### 2.3.1. Differential Scanning Calorimetry (DSC)

The thermal behaviors of PBAT/GMS and PBAT/GTS blends were assessed with differential scanning calorimetry (DSC250, TA Instruments, New Castle, DE, USA). Each 3–5 mg sample sealed in an aluminum pan was heated at a constant rate of 10 °C/min under nitrogen flow (50 mL/min).

The percentage of crystallinity was calculated using the following formula:

(2) $$\chi_{c}=\frac{\Delta H_{m}}{w\times {∆H}_{m}^{\infty }}$$

where $\Delta H_{m}$ is the melting enthalpy of the sample, ${∆H}_{m}^{\infty }$ is the melting enthalpy of 100% crystalline PBT (114 J/g [25]), and w is the percentage of PBAT (wt%) in the sample.

#### 2.3.2. X-Ray Diffraction (XRD)

X-ray diffraction measurements of the films were performed on an Rigaku 3 KW Smartlab diffractometer (Tokyo, Japan) using Cu Kα radiation (λ = 0.15405 nm). The operating voltage and current of the light source were 40 kV and 30 mA, respectively. The diffraction measurements were started at 5° and ended at 60° with a scanning speed of 10°/min.

#### 2.3.3. Dynamic Mechanical Thermal Analysis (DMTA)

Dynamic mechanical thermal analysis (DMTA) measurements were conducted using a TA Instruments DMTA Q-850 with a single cantilever clamp. Then, 400 μm thick films were tested in temperatures sweeping from −60 to 60 °C at a heating rate of 3 °C/min. Experiments were performed in a single frequency oscillation mode at a frequency of 1 Hz under a fixed deformation strain of 0.1%.

#### 2.3.4. Scanning Electron Microscopy (SEM)

A JEOLJSM-7401F scanning electron microscope (Tokyo, Japan) was used to observe the morphology of the cryo-fractured samples. After being sputter-coated with a layer of gold, the fracture surfaces were observed in the high vacuum mode at an accelerating voltage of 10 kV.

#### 2.3.5. Water Vapor Permeability

The water vapor permeability (WVP) of films was calculated using a standard cup test, which was based on the method regulated in ASTM E96 [26]. Circular films of 350–400 μm thick were pre-conditioned in a desiccator for 12 h and then placed on the top of the aluminum cups; they were further sealed with silicone grease at 38 °C and 90 %RH (90% relative humidity). The weight of each cup was recorded during fixed 12 h or 48 h time intervals. The water vapor barrier properties were evaluated using two parameters: water vapor permeability (WVP, g·cm/cm^2^·s·Pa) and water vapor transmission rate (WVTR, g/m^2^·24 h). The WVTR and WVP were derived from Equations (3) and (4):

(3) $$WVTR=\frac{24\cdot ∆m}{A\cdot t}$$

(4) $$WVP=1.157\times 10^{-9}\times \frac{WVTR\cdot L}{∆P}$$

where ∆m (g) is the weight change in the cup within the tested time scale, *A* (m^2^) is the square of the sample, *t* (h) is the time interval after the system stabilizes, *L* (cm) is the thickness of the film, and ∆P (Pa) is the difference in water vapor partial pressure between the two sides of the film.

#### 2.3.6. Contact Angle

Contact angle measurements were performed with contact angle system apparatus (HARKE-SPCAX3, Beijing HARKE Instruments, Beijing, China). Then, 2 μL of distilled water was dropped onto the sample’s surface at room temperature. Images were captured immediately after the drop’s deposition, and the contact angle was recorded using the baseline circle method.

#### 2.3.7. Tensile Measurements

The tensile properties of PBAT/modifiers systems were measured using a universal testing machine (Instron-5967, Norwood, MA, USA). The shape of specimen is a dumbbell of type 5A, which is specified in GB/T 1040.2-2022 [27]. Tests were performed at room temperature and at a tensile rate of 50 mm/min.

### 2.4. Theoretical Methods

Calculations based on molecular dynamics (MD) methods are widely used to assess intermolecular interactions [28,29,30,31]. Here, a molecular dynamic simulation was employed to study the intermolecular interactions between PBAT and GMS or GTS; therefore, the relationship between them and the microstructure was established. The simulations were performed with Materials Studio 2019 software (Biovia Inc., San Diego, CA, USA). The simulated amorphous cells of PBAT, GMS, and GTS were built (details of the molecular models are listed in Table S1); then, geometry optimization and annealing were performed to obtain the lowest energy configuration. The optimized configurations were then used for dynamics simulations under 3000-picosecond NVT and NPT ensembles.

The COMPASS Ⅱ force field is commonly used in the simulation of condensed phase materials; herein, the COMPASS Ⅱ force field was used [28]. The smart algorithm, Andersen control, Berendsen control, and atom-based methods were adopted for geometry optimization, temperature control, pressure control, and electrostatic and non-bonded interactions, respectively. The solubility parameters of PBAT, GMS, and GTS were calculated to analyze compatibility and intermolecular interactions.

## 3. Results and Discussion

### 3.1. Thermal Properties and Crystallinity

**Figure 1:**
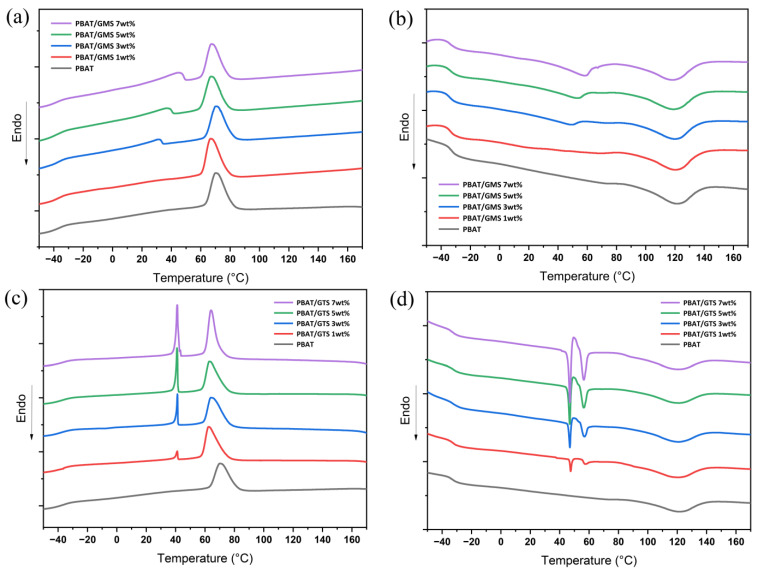
DSC thermograms of PBAT and PBAT with different contents of GMS or GTS: (**a**) first cooling curve of PBAT/GMS, (**b**) second heating curve of PBAT/GMS, (**c**) first cooling curve of PBAT/GTS, and (**d**) second heating curve of PBAT/GTS.

The glass transition temperature ($T_{g}$) and melting enthalpy (${∆H}_{m}$) of polymer materials, which reflect the mobility of the segment and crystallinity of the polymer matrix, respectively, play a crucial role in barrier properties. Representative DSC plots are depicted in Figure 1, while the $T_{g}$, $T_{m}$, crystallization temperature ($T_{c}$) during cooling, melting enthalpy (${∆H}_{m}$), and calculated crystallinity $\left(X_{c}\right)$ values of PBAT are summarized in Table 1. Neat PBAT exhibits a glass transition temperature and melting point of −32.6 °C and 121.0 °C, respectively, which are close to the values reported in the literature [3,8]. The incorporation of both GMS and GTS results in a negligible change in melting point, indicating that the crystallization of PBAT is barely affected. Furthermore, the glass transition temperature, reflecting chain mobility and free volume, affects the gas permeation pathway and thus the barrier property substantially. The mixtures containing GMS and GTS both show a slight change in $T_{g}$, but the changes level off with the addition of more modifiers according to the DSC sand DMA plots (listed in Table 1). This may be connected to the solubility limit of modifiers in PBAT since GMS or GTS molecules quickly reached saturation in the amorphous phase of PBAT, and the superfluous modifiers tend to aggregate and form phase separation; thus, only a small amount is dissolved, resulting in the difference in the $T_{g}$ of PBAT.

For the modifiers, the introduction of GMS into PBAT changes the crystallization temperature and melting point of GMS to some extent, which results from the mixing of GMS and PBAT, thus influencing their crystallization process. The $T_{c}$ of GMS increases from 32.1 °C to 46.2 °C when the concentration of GMS increases from 3 wt% to 7 wt%. On the other hand, PBAT/GTS systems demonstrate a different trend, where $T_{c}$ and $T_{g}$ do not change with the augmentation of the feeding percentages of GTS. Moreover, the crystalline and melting peaks become spiculate and grow with an increase in concentration. During the second heating run, two melting peaks of GTS can be observed in Figure 1c. This may result from two different crystal forms (see Figure S3b) due to insufficient crystallinity in the cooling ramp and because GTS experienced melting and then re-crystallizing processes in the heating ramp, forming a more stable β’ type or α/β’mixed crystal form from α [32]. This variation reflects the different compatibilities of GMS and GTS with PBAT.

**Figure 2:**
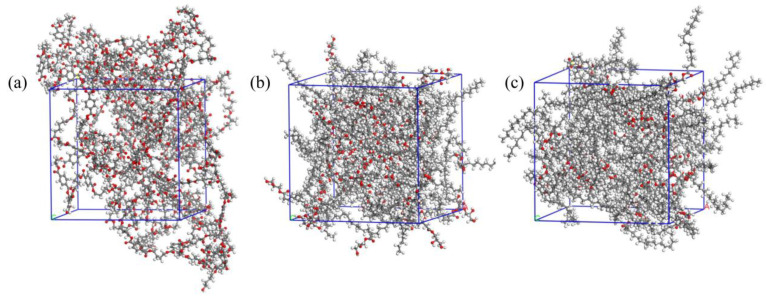
View of the amorphous cells of PBAT (**a**), GMS (**b**), and GTS (**c**) built in Materials Studio. (Red balls denote oxygen atom, grey and white balls represent carbon and hydrogen atoms respectively).

The solubility parameter (σ) is often used to estimate the compatibility between two components, which is affected by bond polarity in the polymer chain. Miscibility is possible when the solubility parameters of two components ($\sigma_{1}$ and $\sigma_{2}$) are close to each other. The solubility parameters were calculated from cohesive energy using molecular dynamics simulation, and the amorphous cells are shown in Figure 2. The calculated σ values of the blend components are as follows: for PBAT, 19.6 MPa^0.5^, which is close to the value of 21.9 MPa^0.5^ reported in the literature; for GMS, 20.2 MPa^0.5^; and for GTS, 17.5 MPa^0.5^. The difference in the solubility parameters of PBAT and GMS is 0.6 MPa^0.5^, while that of PBAT and GTS is 2.1 MPa^0.5^. Taking these results into account, GMS exhibits better compatibility with PBAT, while GTS molecules tend to form phase separation in PBAT due to poor compatibility, which is consistent with the DSC results.

### 3.2. Crystal Structure Analysis

**Figure 3:**
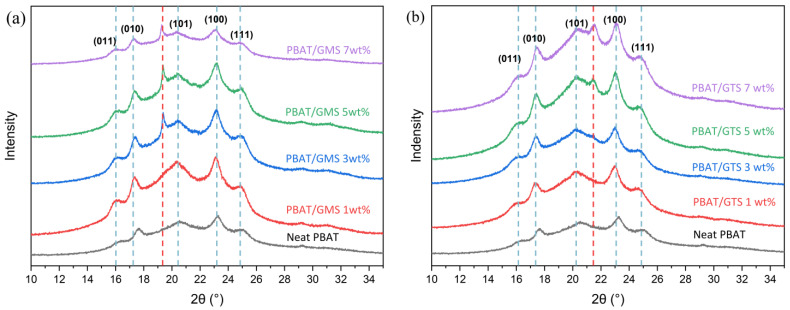
X-ray diffractograms of PBAT and PBAT with different GMS (**a**) or GTS (**b**) contents.

X-ray diffraction was conducted to determine whether the addition of modifiers could influence the crystal structure of PBAT or form co-crystals, and the diffractograms of PBAT/GMS and PBAT/GTS mixtures are shown in Figure 3. PBAT typically shows five diffraction peaks at 2θ values of 16.0°, 17.4°, 20.4°, 23.2°, and 24.9°, indicating the diffraction of the (011), (010), (101), (100), and (111) planes of α-form triclinic crystals, respectively [33,34]. The modifiers GMS and GTS show a single, spiculate peak (denoted with a red dash line), and they do not change the position of original PBAT peaks during the diffraction; this confirms that the incorporated modifiers accumulate in the amorphous phase and do not influence the crystal structure of PBAT.

### 3.3. Thermomechanical Properties

**Figure 4:**
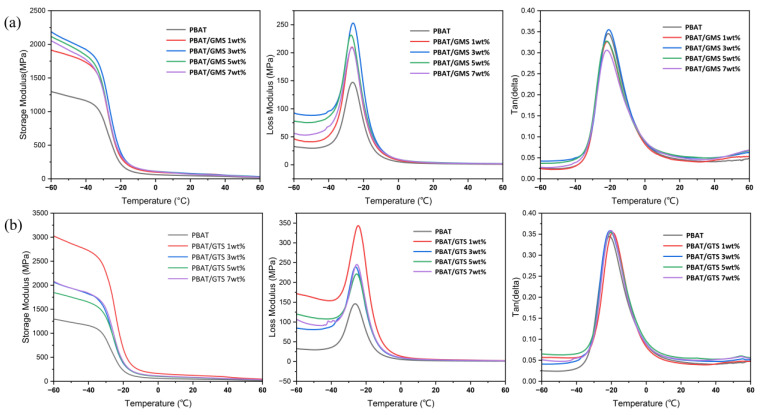
Storage modulus, loss modulus, and tan⁡δ of PBAT and PBAT with different (**a**) GMS and (**b**) GTS contents.

The storage modulus ($E^{\prime }$), loss modulus ($E^{\prime \prime }$), and dissipation factor (tan⁡δ) curves of PBAT and its mixtures are depicted in Figure 4. The storage modulus of neat PBAT exhibits a classic curve with an increase in storage modulus when the temperature increases, and it drops sharply below 1000 MPa near the glass transition temperature, at which point the chain segments of PBAT begin to move cooperatively, which is consistent with the curve reported in the literature [18]. The addition of GMS and GTS increased both the $E^{\prime }$ and $E^{\prime \prime }$ of PBAT to varying degrees below the glass transition temperature, which is attributed to the “stiffening effect” of crystalline modifiers. As discussed by Artur Rozanski et al., the modifiers incorporated into a polymer matrix tend to permeate and accumulate in the interfibrillar amorphous phase since it does not lead to a significant change in the interlamellar distance [21,22]. Therefore, it is the crystal of modifiers in the amorphous phase that causes an increase in the $E^{\prime }$ and $E^{\prime \prime }$ of PBAT mixtures. Furthermore, PBAT mixtures with GMS or GTS exhibit negligible shifts in glass transition temperatures (the peak temperature of tan⁡δ), which denotes the little changes in polymer chain mobility.

### 3.4. Barrier Properties

**Figure 5:**
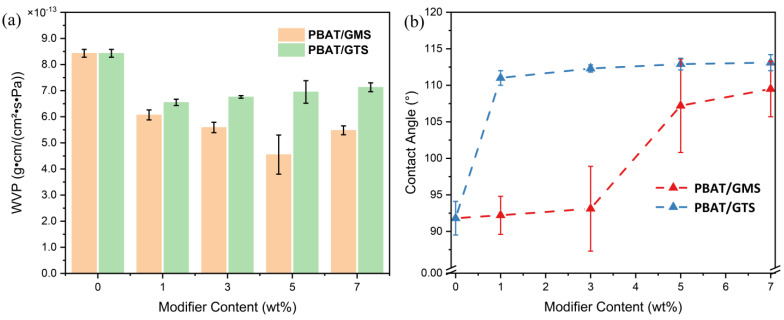
Water vapor permeability and contact angle of PBAT and its blends with different modifiers: (**a**) water vapor permeability recorded after 36 h from the moment of the sample’s preparation; (**b**) contact angle recorded after the sufficient relaxation of samples.

The water vapor barrier property is a crucial consideration for applications in mulching films. As already demonstrated, water vapor permeability is affected by crystallinity, free volume, and surface hydrophobicity. Moreover, in this case, the incorporation of small molecular modifiers barely influences the crystallinity of PBAT, which indicates that the change in barrier properties is primarily determined by the amorphous phases. For PBAT/modifiers systems, modifier content, compatibility, and dispersion are vital factors influencing permeability. In Figure 5a, which presents water vapor permeability columns, it is noted that the WVP decreases first and then increases with an increase in modifier contents, which may have the following explanation: hydrophobicity (solubility of small molecules) declined with an increase in free volume when more modifiers were added. Therefore, at lower concentrations, the hydrophobicity effect was more vital than the increase in free volume introduced by the small molecular modifiers, resulting in lower overall permeability. With a further increase in modifier contents, more free volume was imported and chain mobility was enhanced substantially, which resulted in an increase in permeability. In particular, the WVP was reduced to quite a low value of 4.51 × 10^−13^ g·cm/(cm^2^·s·Pa) when 5 wt% GMS was added to PBAT, which is substantially lower than the 8.43 × 10^−13^ g·cm/(cm^2^·s·Pa) value of neat PBAT. This water vapor barrier exhibits a more significant effect than that of adding more than 10 wt% talc, MXene, and some other inorganic fillers [19,35]. Simultaneously, the WVP decreased slightly to 6.55 × 10^−13^ g·cm/(cm^2^·s·Pa) upon 1 wt% addition and increased gradually to 7.13 × 10^−13^ g·cm/(cm^2^·s·Pa) when more GTS was added. The striking difference in PBAT/GMS and PBAT/GTS systems in terms of water vapor barrier properties comes from the different aggregation states of modifiers in PBAT. As discussed in Section 3.1, glycerol monostearate and PBAT have a narrower solubility parameter difference, meaning better compatibility between the two. In contrast, GTS, due to insufficient compatibility with PABT, promotes phase separation at a scale of 5–10 μm, which will be discussed later in Section 3.6. GTS agglomerates in the PBAT matrix, increasing defects in the PBAT/GTS system; this results in the easy penetration of water vapor or gas molecules into the mixture’s film at the interface, resulting in less desirable barrier properties than PBAT/GMS mixtures.

**Figure 6:**
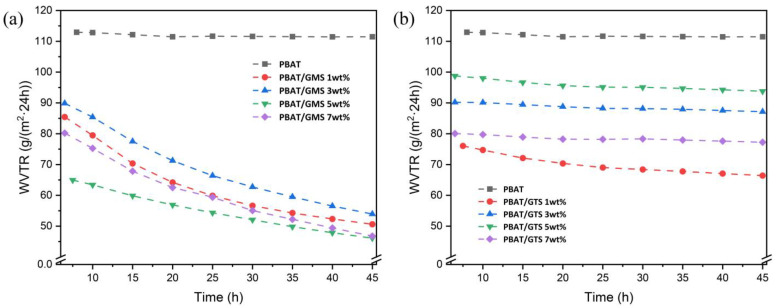
Time-dependent water vapor permeability of PBAT and its blends with (**a**) GMS and (**b**) GTS.

It is worth mentioning that the water vapor transmission rate (or WVP) of PBAT/GMS systems is time-dependent, as shown in Figure 6a. All mixtures with GMS exhibited a declining trend within the recorded timescale, while the water vapor transmission rate of neat PBAT fluctuated at 111.5 g/(m^2^·24 h). In the case of systems with 5 wt% modifier contents, the water vapor permeability decreased notably from 4.51 × 10^−13^ g·cm/(cm^2^·s·Pa) to 2.63 × 10^−13^ g·cm/(cm^2^·s·Pa) after 60 h of relaxation. These changes were also found in PLA/modifier systems [22], and these results were primarily due to the aging process of modifiers. For the aging process, on the one hand, the testing environment (38 °C) contains small molecules that exhibit sufficient homogenizing and crystallizing, which resembles the annealing process of small molecules. On the other hand, the migration of small molecules to the surface of the film permits the formation of hydrophobic microstructures on surfaces, rendering the dissolution of water vapor difficult. Moreover, the migration does not driven by concentration gradient, but rather the phase separation on the surface. In contrast, PBAT/GTS systems presented a rather stable WVTR within the tested time period mainly because glycerol tristearate molecules quickly accumulate due to fast phase separation.

In this study, the effect of reducing WVP through a decrease in solubility is more remarkable than the oxygen permeability (Table 2). This is attributed to the different polarities of water vapor and oxygen. The water vapor of higher polarity is more susceptible to solubility than oxygen, resulting in a more noticeable enhancement in water vapor barrier properties.

### 3.5. Tensile Properties

**Figure 7:**
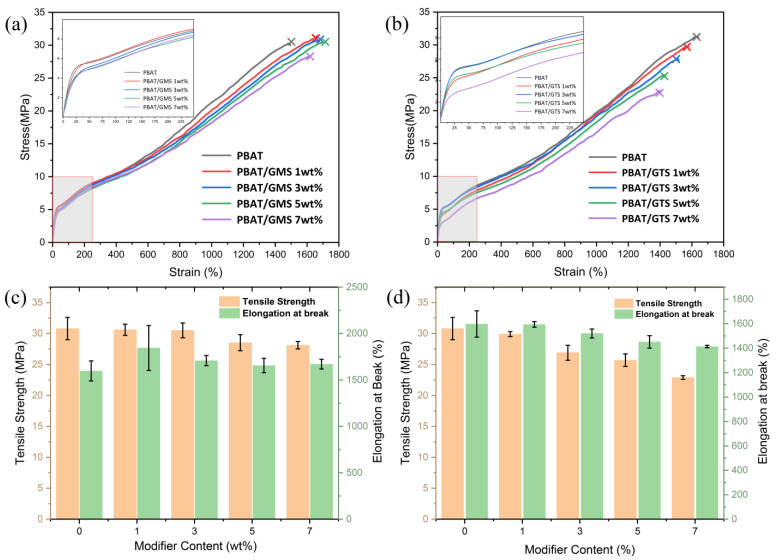
Mechanical properties of PBAT and PBAT/modifiers systems: the tensile curve of PBAT and PBAT blends with GMS (**a**) and GTS (**b**), and the tensile strength and elongation at break of PBAT/GMS (**c**) and PBAT/GTS (**d**).

The mechanical behavior of polymers is essential for mulching film applications, among many other properties. Therefore, the effect of modifiers on the mechanical properties of the PBAT was measured via tensile tests. All samples show the typical tensile characteristics of ductile materials, among which neat PBAT shows the highest tensile strength of 30.9 MPa, with an elongation at break of 1598% (Figure 7). The tensile strength of the mixtures was reduced to varying degrees with different additions of GMS and GTS, while the elongation at break did not change significantly. The addition of GMS to PBAT resulted in a slight decrease in mechanical strength from 30.9 MPa for neat PBAT to 28.1 MPa for the maximum addition. However, this decline in tensile strength is negligible compared to the compositions of inorganic fillers [19,34]. GTS, due to its insufficient compatibility with PBAT, reduced the homogeneity of the polymer matrix and formed defects, which hindered stress transfer during stretching [34], resulting in a marked decrease in the tensile strength and elongation at break of the PBAT/GTS mixtures.

### 3.6. Fracture Surface and Surface Hydrophobicity

**Figure 8:**
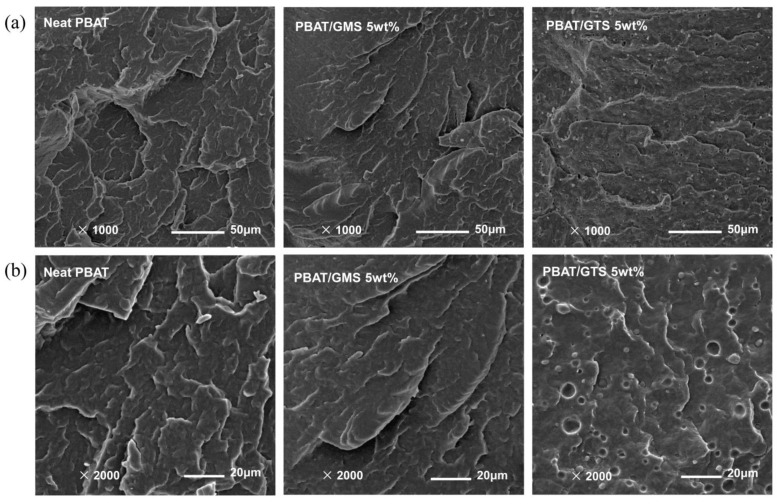
SEM micrographs of the cryo-fractured surfaces of PBAT and PBAT blends with 5 wt% GMS (**a**) or GTS (**b**).

Figure 8 shows the scanning electron microscopy (SEM) micrographs for the cryo-fractured surfaces of some representative morphologies, revealing an obvious difference between PBAT blends with GMS or GTS. In the cross surface of PBAT with 5 wt% GMS, no phase separation was observed, while the cross surface with 5 wt% GTS showed a noticeable phase separation structure. From this perspective, it is explained that GTS is less compatible with PBAT and the phase interface is defective, allowing the passage of gas or water vapor molecules; this is also supported by the solubility parameters calculated via molecular dynamic simulation.

The contact angle test was carried out after the sufficient relaxation of samples. The surface hydrophobicity of films is shown in the contact angle (CA) values (Figure 5b), which agrees with the trend in water vapor permeability. As discussed in Section 3.4, the best water vapor barrier properties appeared when 5 wt% GMS or 1 wt% GTS was added, where the contact angles reached the highest values.

## 4. Conclusions

The effects of glycerol monostearate and glycerol tristearate on the thermal, mechanical, and water vapor properties of PBAT were investigated via experiments and simulations. The addition of modifiers slightly influenced the melting point and glass transition temperature according to the DSC and DMTA tests. It was verified that the addition of GMS and GTS did not change the crystal structure of PBAT, which is advantageous for only tailoring the amorphous phase of PBAT. The water vapor barrier property, as the focus of this study, was improved first and then reduced afterward with the addition of modifiers, reaching optimal WVP values of 2.63 × 10^−13^ g·cm/(cm^2^·s·Pa) and 6.55 × 10^−13^ g·cm/(cm^2^·s·Pa) when 5 wt% GMS and 1 wt% GTS were added, respectively; these values are substantially lower than the 8.43 × 10^−13^ g·cm/(cm^2^·s·Pa) WVP value for neat PBAT. This improvement in WVP is assumed to be related to the enhancement of hydrophobicity on the surface of the modified PBAT films, agreeing with the corresponding changes in the contact angle. In addition, the water barrier properties were enhanced with the relaxation of the samples, which is explained by the balancing and migration of modifiers. We should emphasize that the incorporation of glycerol monostearate modifiers did not substantially sacrifice the mechanical properties of PBAT. Our results indicate that the water vapor permeability was tightly connected with the content, polarity, and compatibility of modifiers within the polymer matrix.

## Figures and Tables

**Table 1:** Thermal properties of PBAT and PBAT with small molecular modifiers (PBAT/GMS and PBAT/GTS) obtained via DSC and DMA.

Samples	$T_{c}$ (°C)	$T_{g}$ (°C) ^a^	$T_{g}$ (°C) ^b^	$T_{m}$ (°C)	${∆H}_{m}$ (J/g)	$X_{c}$ (%)
Neat PBAT	70.3	−32.6	−21.1	121.0	12.8	11.2
PBAT/GMS 1 wt%	66.7	−33.2	−21.8	120.6	12.9	11.4
PABT/GMS 3 wt%	70.3	−34.5	−20.8	120.2	12.2	11.0
PABT/GMS 5 wt%	67.0	−34.0	−21.9	119.2	13.0	12.0
PBAT/GMS 7 wt%	67.4	−34.3	−21.9	118.4	11.2	10.6
PABT/GTS 1 wt%	62.7	−31.6	−19.3	119.6	17.3	15.3
PABT/GTS 3 wt%	64.1	−30.9	−20.8	120.1	13.3	12.0
PABT/GTS 5 wt%	63.1	−31.3	−20.3	120.3	14.6	13.5
PABT/GTS 7 wt%	64.2	−31.6	−20.3	119.3	16.8	15.8

$T_{g}$^a^ and $T_{g}$ ^b^ were obtained via DSC and DMA, respectively. The crystallinity of PBAT ($X_{c}$) was calculated using the melting enthalpy of 100% crystalline PBT.

**Table 2:** Oxygen and water vapor permeability for PBAT blends with modifiers.

Samples	P(H_2_O, 36 h)	BIF	P(H_2_O, 60 h)	BIF	P(O_2_)	BIF
Neat PBAT	8.43 × 10^−13^	1.0	8.43 × 10^−13^	1.0	2.87 × 10^−13^	1.0
PBAT/GMS 1 wt%	6.07 × 10^−13^	1.4	3.59 × 10^−13^	2.3	2.57 × 10^−13^	1.1
PABT/GMS 3 wt%	5.59 × 10^−13^	1.5	4.10 × 10^−13^	2.1	2.72 × 10^−13^	0.9
PABT/GMS 5 wt%	4.51 × 10^−13^	1.9	2.63 × 10^−13^	3.2	2.67 × 10^−13^	1.0
PBAT/GMS 7 wt%	5.48 × 10^−13^	1.5	3.43 × 10^−13^	2.5	2.44 × 10^−13^	1.1
PABT/GTS 1 wt%	6.55 × 10^−13^	1.3	4.77 × 10^−13^	1.8	2.61 × 10^−13^	1.1
PABT/GTS 3 wt%	6.76 × 10^−13^	1.2	6.88 × 10^−13^	1.2	2.71 × 10^−13^	0.9
PABT/GTS 5 wt%	6.95 × 10^−13^	1.2	7.16 × 10^−13^	1.2	3.07 × 10^−13^	0.9
PABT/GTS 7 wt%	7.13 × 10^−13^	1.2	5.76 × 10^−13^	1.5	2.99 × 10^−13^	1.0

P(H_2_O, 36 h) and P(H_2_O, 60 h) were recorded after 36 h and 60 h from the moment of sample preparation. The values P(O_2_) and P(H_2_O) are united as cm^3^·cm/(cm^2^·s·Pa) and g·cm/(cm^2^·s·Pa), respectively. BIF is defined as the permeability of neat PBAT divided by PBAT/modifier blends.

## Data Availability

The original contributions presented in this study are included in the article/Supplementary Materials. Further inquiries can be directed to the corresponding authors.

## Supplementary Materials

The following supporting information can be downloaded at https://www.mdpi.com/article/10.3390/ma17235732/s1. Figure S1: Oxygen permeability of PBAT and its blends with different modifiers; Figure S2: DSC thermograms of GMS (a) and GTS (b); Figure S3. X-ray diffractograms of GMS (a) and GTS (b); Table S1: Details of the molecular models used in this work and calculated solubility parameters of different samples; Table S2: Contact angle of PBAT and its blends with different modifiers (data were recorded after the sufficient relaxation of samples). References [29,30,32,34,36] are cited in the supplementary materials.
