# Supplementary material for: Effect of Glycerol Stearates on the Thermal and Barrier Properties of Biodegradable Poly(butylene Adipate-Co-Terephthalate)
Source: Materials (Basel). 2024 Nov 23;17(23):5732. doi: 10.3390/ma17235732 (PMC11642522; doi:10.3390/ma17235732)
Supplement: Supplementary file 1 [file materials-17-05732-s001.zip › materials-3316434-supplementary.pdf]

Supporting Information

Effect of Glycerol Stearates on the Thermal and Barrier Properties of Biodegradable Poly(butylene Adipate-co-terephthalate)

Jing Yuan [1], Xinpeng Zhang [1], Jun Xu [1,*], Jianping Ding [2], Wanli Li [2] and Baohua Guo [1,*]

[1] Key Laboratory of Advanced Materials (MOE), Department of Chemical Engineering, Tsinghua University, Beijing 100084, China; yuanj22@mails.tsinghua.edu.cn (J.Y.); zhangxp20@mails.tsinghua.edu.cn (X.Z.)

[2] Xinjiang Blue Ridge Tunhe Sci. & Tech. Co., Ltd., Changji 831100, China

* Correspondence: jun-xu@mail.tsinghua.edu.cn (J.X.); bhguo@mail.tsinghua.edu.cn (B.G.)

Table S1. Details of the molecular models used in this work and calculated solubility parameters of different samples.

Samples	Number of repeat units	Chains	Cell volume (Å^3)	Solubility Parameter ((J/cm^3)$^{0.5}$)	Solubility Parameter in reference ((J/cm^3)$^{0.5}$)
PBAT	40	4	48784.4	19.6	21.9[1]
GMS	1	60	50437.2	20.2	-
GTS	1	40	49463.2	17.5	-

Table S2. Contact angle of PBAT and its blends with different modifiers (data were recorded after the sufficient relaxation of samples).

Samples	Contact Angle (°)
Neat PBAT	91.8±2.3
PBAT/GMS 1wt%	92.2±2.6
PABT/GMS 3wt%	93.1±7.1
PABT/GMS 5wt%	107.2±6.4
PBAT/GMS 7wt%	109.5±3.8
PABT/GTS 1wt%	111.0±0.4
PABT/GTS 3wt%	112.3±0.5
PABT/GTS 5wt%	112.9±0.8
PABT/GTS 7wt%	113.1±1.1

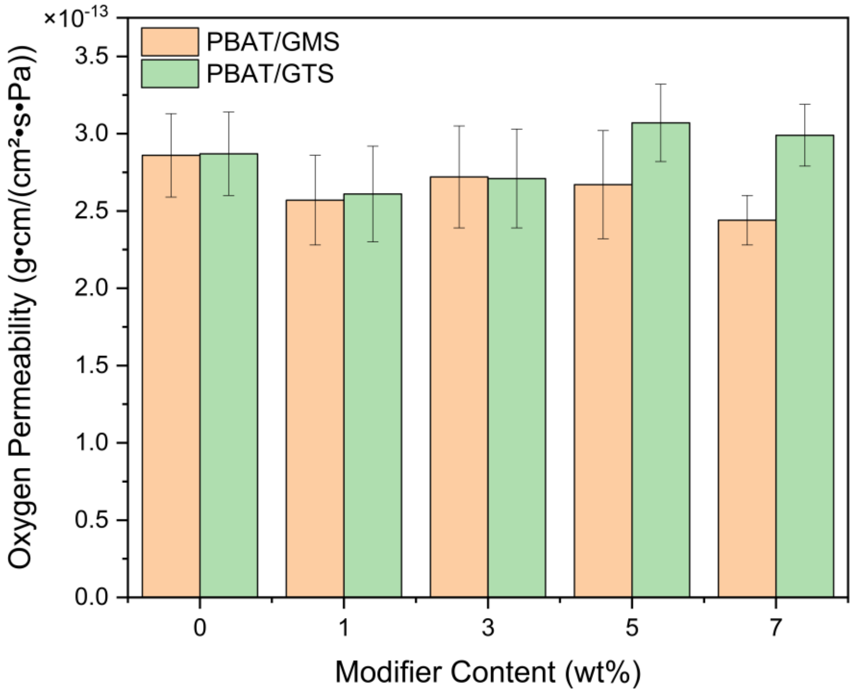

Figure S1. Oxygen permeability of PBAT and its blends with different modifiers.

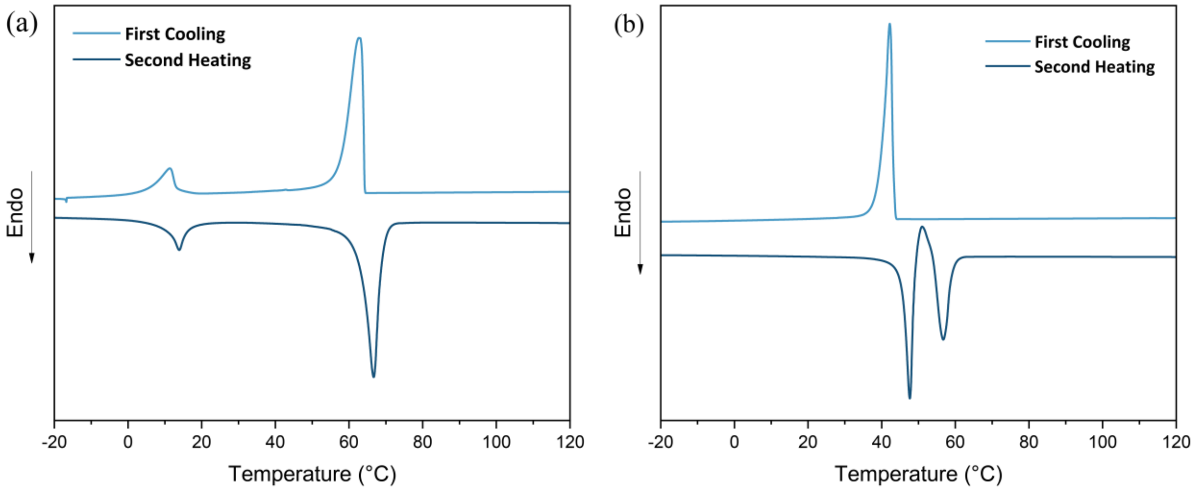

Figure S2. DSC thermograms of GMS (**a**) and GTS (**b**).

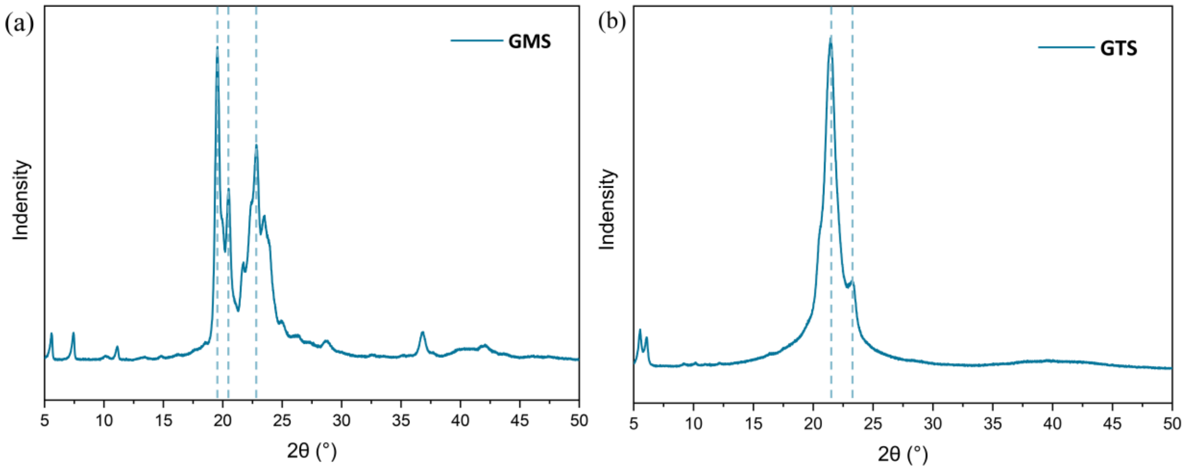

Figure S3. X-ray diffractograms of GMS (**a**) and GTS (**b**).

References

1. Su, S.; Duhme, M.; Kopitzky, R. Uncompatibilized PBAT/PLA Blends: Manufacturability, Miscibility and Properties. *Materials* **2020**, *13*, 4897. https://doi.org/10.3390/ma13214897.
2. Sato, K.; Ueno, S. Crystallization, transformation and microstructures of polymorphic fats in colloidal dispersion states. *Curr. Opin. Colloid Interface Sci.* **2011**, *16*, 384–390. https://doi.org/10.1016/j.cocis.2011.06.004.
3. Pulikkalparambil, H.; Phothisarattana, D.; Promhuad, K.; Harnkarnsujarit, N. Effect of silicon dioxide nanoparticle on microstructure, mechanical and barrier properties of biodegradable PBAT/PBS food packaging. *Food Biosci.* **2023**, *55*, 103023. https://doi.org/10.1016/j.fbio.2023.103023.
4. Zhao, M.; Zhang, C.L.; Yang, F.; Weng, Y.X. Gas barrier properties of furan-based polyester films analyzed experimentally and by molecular simulations. *Polymer* **2021**, *233*, 124200. https://doi.org/10.1016/j.polymer.2021.124200.
5. Tian, S.A.; Cao, X.Z.; Luo, K.Q.; Lin, Y.Y.; Wang, W.J.; Xu, J.; Guo, B.H. Effects of Nonhydroxyl Oxygen Heteroatoms in Diethylene Glycols on the Properties of 2,5-Furandicarboxylic Acid-Based Polyesters. *Biomacromolecules* **2021**, *22*, 4823–4832. https://doi.org/10.1021/acs.biomac.1c01106.
